# Synthesis and Antibacterial Activities of Novel 4-Hydroxy-7-hydroxy- and 3-Carboxycoumarin Derivatives

**DOI:** 10.3390/molecules170910846

**Published:** 2012-09-10

**Authors:** Pen-Yuan Lin, Kuang-Sheng Yeh, Chien-Ling Su, Shiow-Yunn Sheu, Tiffany Chen, Keng-Liang Ou, Mei-Hsiang Lin, Lin-Wen Lee

**Affiliations:** 1 School of Pharmacy, College of Pharmacy, Taipei Medical University, 250 Wuxing St., Taipei 11031, Taiwan; 2 Department of Veterinary Medicine, School of Veterinary Medicine, College of Bioresources and Agriculture, National Taiwan University, No. 1, Sec. 4, Roosevelt Rd., Taipei 10617, Taiwan; 3 School of Respiratory Therapy, Taipei Medical University, Taipei Medical University, 250 Wuxing St., Taipei 11031, Taiwan; 4 Department of Biochemistry and Chemistry, University of Washington, 4311 11th Ave. NE, Seattle, WA 98105-4068, USA; 5 Research Center for Biomedical Devices and Prototyping Production, Taipei Medical University, 250 Wuxing St., Taipei 11031, Taiwan; 6 Department of Microbiology and Immunology, School of Medicine, College of Medicine, Taipei Medical University, 250 Wuxing St., Taipei 11031, Taiwan

**Keywords:** 4-hydroxy-, 7-hydroxy-, 3-carboxycoumarin derivatives, antibacterial activities

## Abstract

Coumarin derivatives are used as fluorescent dyes and medicines. They also have some notable physiological effects, including the acute hepatoxicity and carcinogenicity of certain aflatoxins, the anticoagulant action of dicoumarol, and the antibiotic activity of novobicin and coumerymycin A1. Because the number of drug resistant strains is increasing at present, the synthesis of new antibacterial compounds is one of the critical methods for treating infectious diseases. Therefore, a series of coumarin-substituted derivatives, namely 4-hydroxy- and 7-hydroxycoumarins, and 3-carboxycoumarins were synthesized. 4-Hydroxycoumarin derivatives **4a–c** underwent rearrangement reactions. Both 4- and 7-hydroxycoumarins were treated with activated aziridines which produced series of ring-opened products **7**, **8**, **10**, and **11**. 3-Carboxy-coumarin amide dimer derivatives **14–21** were prepared by reacting aliphatic alkylamines and alkyldiamines with PyBOP and DIEA. In this study, we use a new technique called modified micro-plate antibiotic susceptibility test method (MMAST), which is more convenient, more efficient, and more accurate than previous methods and only a small amount of the sample is required for the test. Some of the compounds were produced by reactions with acid anhydrides and demonstrated the ability to inhibit Gram-positive microorganisms. The dimer derivatives displayed lower antibacterial activities.

## 1. Introduction

Coumarin is a lactone compound which is widely distributed in plants and can be extracted, among others, from *Anthoxanthum odoratum*, *Melilotus officinalis*, *Dipterix oppositifolia* and *Dipterix oppositifolia* [1]. Its derivatives are used as daylight fluorescent pigments [2], brighteners [3], fluorescent dyes [4], fluorescent sensors [5], laser dyes [6], drug carriers [7], and drug delivery agents [8]. Recently, various medicinal effects, such as anticoagulant [9,10], analytical fluorescence indicator, anti-inflammatory [11] and antioxidant [12,13,14,15] properties have been reported. Many authors have reported the convenient and effective synthesis of the dimers from salicylaldehydes and by biomimetic synthesis. Acetoxycoumarins, polycyclic 7-hydroxycoumarins and other coumarin derivatives display anticarcinogenic properties [16,17,18,19,20,21,22]. Roma also reported that tricyclic or bicyclic coumarin derivatives with amino groups showed antiplatelet activity [23]. 4-Heteroarylamino-, triazolopyridine, imidazolopyridine, *N*-substituted-3-carboxamido- and other groups incorporate a coumarin moiety displaying new coumarin derivative structures which showed antibacterial [24,25,26,27,28,29] and antifungal [30] activities. Several novel coumarin analogs possess antimycobacterial activity [31,32,33]. Modification of the chemical structure of the coumarin parent skeleton showed unit enzyme inhibition *in vitro* and *in vivo* [34,35,36,37]. Coumarin also combines with sugar units to form C-glycosyl compounds. In this study, we report the characteristics of several chemicals including 4-hydroxycoumarin derivatives **1a–c**, **5**, and 7-hydroxycoumarin **9** and 3-carboxycoumarin derivatives **12**. By using acid anhydrides, activated aziridines **6**, benzotriazol-1-yloxtri-(pyrrolidinol)phosphonium hexafluorophosphate (PyBOP, **13**), diisopropylethylethylamine (DIEA), dimethylformamide (DMF), alkylamines, and alkyldiamines to react with 3-carboxycoumarin, instead of dicyclohexylcarbodiimide (DCC) as a coupling reagent high yields of 3-carboxycoumarin amide dimers were obtained.

Microbiological trials using the minimal inhibitory concentration (MIC) on penicillin G, amikin and the new modified micro-plate antibiotic susceptibility test (MMAST) were carried out on different bacterial strains to test the activities of the new compounds [38].

## 2. Results and Discussion

Acylation of **1a–c** with acid anhydride in a suitable solvent at ca. 150 °C caused the substitution to produce **3a–c**, **3a_1_–c_1_** and **3a_2_–c_2_**. The acyl groups were removed via rearrangement to produce compounds **4a–c**, **4a_1_–c_1_**, and **4a_2_–c_2_** under heating conditions. 4-Acyloxycoumarins experienced a greater extent of rearrangement than 7-acyloxycoumarins. The effects might have been due to an electron deficiency inductive effect plus the different distances between position 7-acyloxy and the non-aromatic double-bond electron density at positions 3 and 4 of the 4-acyloxycoumarins. In this study, only the aliphatic acid anhydride acylation of 4-hydroxycoumarin caused rearrangements, while aromatic acid anhydrides failed to react. For 7-hydroxycoumarin, its acylation product was not observed, even when aliphatic or aromatic acid anhydrides with NaOMe in methanol were used. When 7-hydroxycoumarin treated with base will become more resonance anion and more difficult to react with anhydride by weak base MeOH and even by strong base NaOMe. Therefore both of bases are unable to carry out the reaction. We also tried to use other diketone and amide compounds to react with the hydroxycoumarins, unfortunately, the expected acylation rearrangement did not occur. This might be explained by the greater number of methylene groups and the increase in the distance between the two C=O bonds which makes it more difficult to attack the other carbonyl position. Synthesis of coumarin derivatives via the ring-opening reaction with activated aziridines has not been reported and characterized previously. The reaction of the coumarin anion with *N*-benzoyl- or *N*-sulfonylaziridines **6** yielded amidoethylated 7- or 4-hydroxycoumarins **7**, **8**, **9** and **10**. The final results depend on the substitution of the *N*-acyl or *N*-sulfonyl groups on the acyl group, such as benzoyl and the coumarin anions will attack from an abnormal position to provide the major products **7d–f**, **10n**, and **10q**. Only trace amounts of products are produced from normal ring-openings of aziridines. However, with the *N*-sulfonyl group, we obtained both products (in 4:1 ratio), but the main products resulted from normal ring-opening of the activated aziridines **7h**, **8i**, **8j**, **10k–l**, and **11k**. It was assumed that compounds **7**, **8**, **10**, **11**, and their analogues were formed by the nucleophilic attack of acylaziridines via an S_N_2-like ring opening mechanism. The comparison of the proton NMR of the NH group in **7** and **8** revealed the cleavage of the ring position. 

In our previous work [35], we reported that when PyBOP (**13**) was used as a coupling reagent to synthesize natural amide dimers, relatively high yields was obtained. The reaction did not perturb the formation of amide dimers even when a sugar moiety is attached to the parent compounds. In general, for the formation of amide or dimer chains, the reaction temperatures had a great influence on the structural conformation. From NMR spectroscopic analysis, the intermediate, coumarin-triazobenzene, displayed rearranged characteristics. We hypothesize that it might depend on the two factors of the solvent system introduced and the duration of the reaction. The reaction was carried out under mild conditions, by mixing the reagents with DMF at ca. 0 °C, followed by the addition of DIEA. The resulting solution was allowed to warm to room temperature for 1–3 days, then alkyldimines were added dropwise to produce high yields of coumarin amide dimers.

All of the above synthetic products were further tested for their antibacterial effects. The MICs of the compounds tested against the reference strains are shown in Table 1. The antimicrobial tests performed on coumarin derivatives **3c**, **3c_1_**, **4a_2_**, **4b**, **4c**, and **7f** confirmed the better activities of these compounds against Gram-positive rather than Gram-negative bacteria and some were especially active against *Bacillus subtilis* and *Staphylococcus aureus*. Compound **7f** was the most potent of the tested compounds against *B. subtilis*, with an MIC value of 8 μg/mL. Compounds **3c**, **3c_1_**, **4a_2_**, **4b**, **4c**, and **7d** only inhibit bacterial growth moderately, with lower MICs of 32, 16, and 64 μg/mL, respectively, when compared to penicillin G. The MICs of coumarin amide dimers **14–21** ranged from 128 to 256. Prior to these studies, the available information on coumarin-aziridins and the dimerization moiety of SAR indicated that molecular recognition involved specific receptor interactions. We propose that the various aziridines and dimer chain lengths might change the binding characteristics of ligands or the conformational direction to their respective receptors and, thereby, improve the biological activities. 

**Table 1:** MICs of compounds **3**, **4**, **7**, **8**, **10**, **11**, and **14–21** against the reference strains.

	MIC (μg/mL) *^a^*			
	Gram (+) bacteria *^b^*		Gram (−) bacteria ^b^	
Compds.	*B. s.*	*S. a.*	*E. c.*	*P. a.*
**3a**	>256	>256	>256	>256
**3a_1_**	>256	>256	>256	>256
**3a_2_**	128	>256	>256	>256
**3b**	128	>256	128	>256
**3b_1_**	128	256	256	>256
**3b_2_**	>256	>256	>256	>256
**3c**	32	32	>256	>256
**3c_1_**	32	>256	>256	>256
**3c_2_**	>256	>256	>256	>256
**4a**	128	>256	>256	>256
**4a_1_**	>256	>256	>256	>256
**4a_2_**	64	>256	>256	>256
**4b**	64	128	256	>256
**4b_1_**	>256	>256	>256	>256
**4b_2_**	128	>256	>256	>256
**4c**	16	16	128	>256
**4c_1_**	>256	>256	>256	>256
**4c_2_**	64	32	>256	>256
**7d**	64	64	>256	>256
**7e**	>256	>256	>256	>256
**7f**	8	8	>256	>256
**7g**	>256	>256	>256	>256
**7h**	>256	>256	>256	>256
**8i**	>256	>256	>256	>256
**8j**	>256	>256	>256	>256
**10k**	>128	>128	128	>128
**10l**	>128	>128	>128	>128
**10m**	>128	>128	128	>128
**10n**	>128	>128	>128	>128
**10o**	>128	>128	>128	>128
**10p**	>128	>128	>128	>128
**10q**	>128	>128	>128	>128
**10r**	>128	>128	>128	>128
**11k**	>128	>128	>128	>128
**14**	>128	>128	>128	>128
**15**	>128	>128	>128	>128
**16**	>128	>128	>128	>128
**17**	>128	>128	>128	>128
**18**	>128	>128	>128	>128
**19**	>128	>128	>128	>128
**20**	>128	>128	>128	>128
**21**	>128	>128	>128	>128
**PenicillinG *^c^***	8	0.0625	-	-
**Amikin**	-	-	1	0.125

*^a^* MIC, minimal inhibitory concentration. *^b^* Bacteria: *B. s., Bacillus subtilis (*BCRC 10029); *S. a., Staphylococcus aureus* (BCRC 11863); *E. c., Escherichia coli* (BCRC 11758); *P.* *a., Pseudomonas aeruginosa* (BCRC 11733). *^c^* Penicillin G potassium salt, Sigma p8721, lot 102K0483, 1,589 units/mg.

### 2.1. Chemistry

**Scheme 1:**
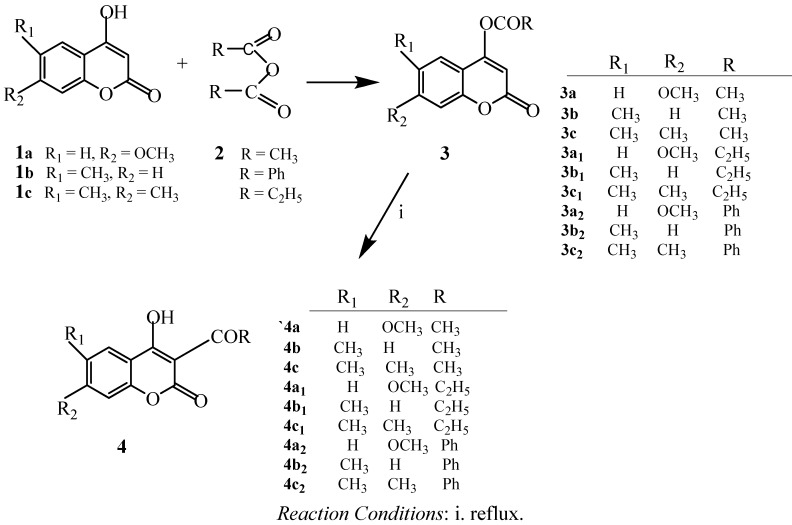
4-Hydroxycoumarin reacted with acid anhydride.

Regarding the synthetic route applied to 4-hydroxycoumarin derivatives, 4-hydroxycoumarin and its analogues **1a–c** were used as starting materials to react with acid anhydrides, acetic anhydride, e.g., propionic anhydride, and benzoic anhydride, to produce the corresponding O-acyloxyl products **3a–c**. It was observed that a proton on the hydroxyl group of coumarin was substituted by an acyl group when we elevated the temperature to ca. 150 °C. Similar conditions were adopted to produce the *O*-acyloxyl and benzoyloxyl derivatives as the main reaction products. High yields were achieved when methyl groups were introduced at the 6 and 7 positions of the parent coumarin structure (Scheme 1).

**Scheme 2:**
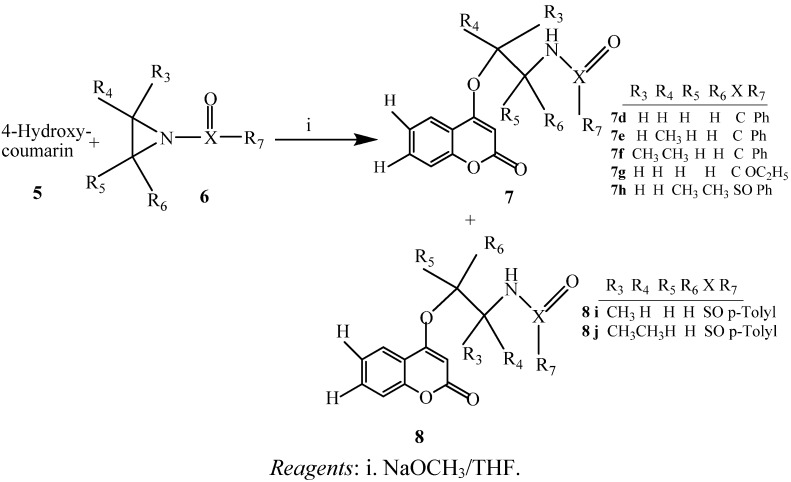
4-Hydroxycoumarin reacted with activated aziridines **6**.

**Scheme 3:**
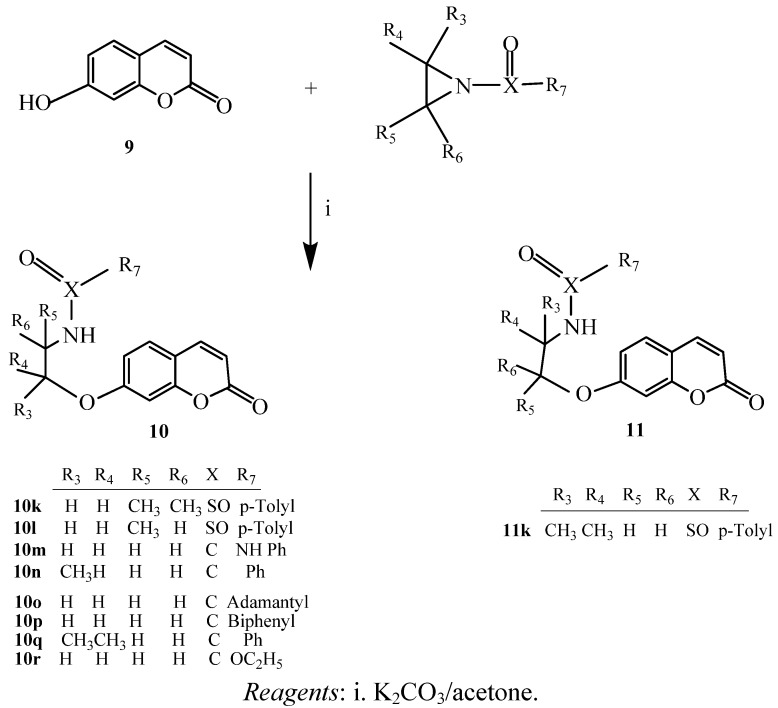
Reactions of 7-hydroxycoumarin with activatived aziridines **6**.

The subsequent reaction involving heating over 170 °C in a sealed tube led to the rearrangement products **4a–c** in yields comparable to compounds **3a–c**. Methyl group substitution produced greater yields of 52%–64%. Excess acid anhydrides were finally removed by column chromatography on silica gel. Furthermore, 4-hydroxy- and 7-hydroxycoumarin derivatives were found to undergo ring-opening reactions with various activated aziridines (Scheme 2 and Scheme 3).

These new and convenient approaches to the synthesis of 4-hydroxy-, 7-hydroxycoumarins or other hydroxycoumarin derivatives were apparently caused by the nucleophilic attack on the activated aziridine rings. We used two hydroxycoumarins as starting materials in refluxing tetrahydrofuran (THF) and acetone in the presence of a base (NaOCH_3_ or K_2_CO_3_) for 2–3 h to form “coumarin 5-O” and “coumarin 9-O” [the anions of 4-hydroxycoumarin (**5**) and 7-hydroxycoumarin (**9**)], and then reacted them with various activated aziridines. It was found that two isomers **7**, **8** or **10**, **11** were produced from each hydroxycoumarin in yields of 28%–88%. The main products generated from *N*-benzoylaziridines (X = C) were produced by abnormal regioselective ring-opening reactions producing **7d–g**, **10m–n**, and **10q** with strongly preferred cleavage of the N–CH_2_ bond. In the similar reactions with tolylaziridines (X = SO), a mixture of normal regioselectivity ring-opening products **7h**, **8i–j**, **10k–l**, and **11k **were observed to predominate.

**Scheme 4:**
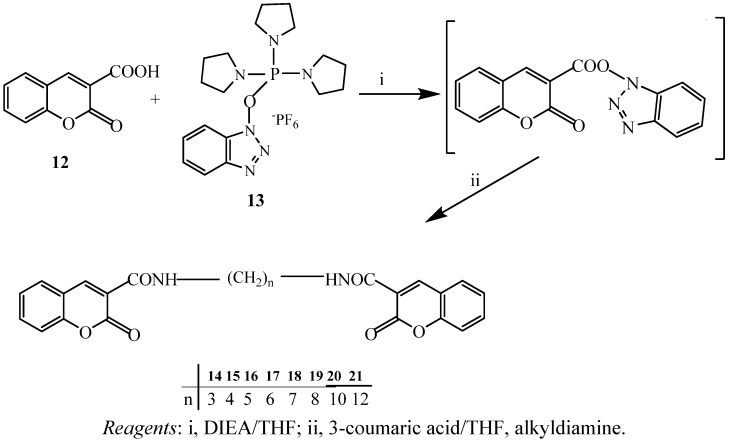
3-Carboxycoumarin reactions with alkyldiamines.

The syntheses of some coumarin dimers using Mn(III) or Fe(III) in the presence of a strong acid to obtain high yields were reported. We reacted coumarin acid **12** with diamines (n = 3–12) in the presence of PyBOP, DIEA, and dimethylformamide at room temperature or 60 °C with stirring for 18 h. The excess solvent, DIEA, and alkyldiamines were removed by column chromatography on silica gel to provide coumarin amide dimers **14–21** in excellent yields (82%–87%). The products were recrystallized from ether and methanol (Scheme 4). The yields of dimers seem not to be affected by the bulky PyBOP reagent and essentially no steric hindrance between diamines and coumarins was observed. The chemical structures of these products were established using spectroscopic methods including IR, ^1^H-, ^13^C-NMR, FAB-MS, HR-FAB-MS, and LC/MS/MS (ESI). The IR spectra of these amide dimers showed the C=O absorption peaks around 1647–1665 cm^−1^. In the ^1^H-NMR (in CDCl_3_) the amide N–H absorption appeared at δ 8.79–8.92 ppm as a broad singlet. The ethylene amide dimers of coumarins revealed doublet of doublet signals at δ 3.47 ppm (–NH–CH_2_–). ^13^C-NMR showed the amide (NH–C) signal at δ 161.4–161.8 ppm in the parent compounds.

### 2.2. Antibacterial Activity Test

Acyl coumarins **3a–4c**, 4-hydroxy-, and 7-hydroxycoumarins **7–11** and coumaric amide dimers **14–21** were tested against reference strains of *Bacillus subtilis* (BCRC 10029), *Staphylococcus aureus* (BCRC 11863), *Escherichia coli* (BCRC 11758), and *Pseudomonas aeruginosa* (BCRC 11733) obtained from The Bioresources Collection & Research Center (BCRC), Taiwan. Penicillin G potassium salt (CAS 113-98-4, USP grade) was used as a reference and purchased from Sigma-Aldrich (St. Louis, MO, USA). Bacteria were grown overnight in Müller-Hinton broth (Difco, Detroit, MI, USA) and trypticase soy broth (TSB, Oxoid, Basingstoke, Hampshire, UK).

The minimal inhibitory concentrations (MICs) of all synthesized compounds were determined using the reference method of broth dilution measurements. Each test compound and penicillin G was dissolved in DMSO/EtOH/phosphate buffer or phosphate buffer before a serial two-fold dilution to the desired test concentration ranges. DMSO/EtOH/phosphate buffer and phosphate buffer were used for the solvent control test. The seed was cultured with trypticase soy broth containing 10^7^ colony forming units (cfu)/mL. Müller-Hinton broth was sterilized by autoclaving at 120 °C for 15 min, and microorganism were suspended in 96-well microplate. The final concentration of microorganism in each well was 10^5^ cfu/mL. Modified 96-well plates were used with a two-fold micro-dilution method of the MTT assay in Müller-Hinton broth. Microorganism suspensions containing 10^5^ cfu/mL were inoculated onto the 96-well microplates and mixed with the test compounds. The MIC was defined as the lowest concentration of test compounds, allowing no visible growth of test strain bacteria after incubation at 37 °C for 3 h. Then SDS in 0.1 N HCl was added. The absorbance was measured by ELISA reader (Bio-Tek Instruments, Microplate Autoreader, EL 311) at 595 nm.

## 3. Experimental

### 3.1. General

The IR spectra were recorded on a Thermo Mattson IR300 and Nicolet 510 PET spectrometers. Optical rotations were measured with a JASCO p-1020 digital polarimeter. NMR spectra were recorded at 500 (^1^H-NMR) and 125 MHz (^13^C-NMR) on a Bruker DRX-500 spectrometer in CDCl_3_ or DMSO solution with respect to the corresponding solvent peak used as the internal standard. Mass spectra were measured on a Finnigan/Thermo Quest NAT 95XL, JEOL JMX-HX 110, JEOL JMSHX 110 FAB-MS, and LC/MS/MS Micromass Quattro II. The compounds were prepared according to similar procedures, and reactions took place in high-pressure tubes (Büchiglasuster, bursting disc, 0032). For the chromatographic analysis, Merck silica gel 60 was used. The chemical reagents used in the synthesis were purchased from Sigma-Aldrich and Merck. The crude products were purified by column chromatography and recrystallized using ether/methanol or *n*-hexane/methanol. Biological data were recorded on a Microplate Autoreader EL 311.

### 3.2. Preparation of Acylcoumarins

4-Hydroxycoumarin and its derivatives **1a–c** were added to a high-pressure tube (Bursting disc, Büchiglasuster) containing of acid anydride **2**. The solution was stirred for 2 h at 150 °C. The dried mass was separated by column chromatography (CHCl3/EA/n-hexane = 8:1:1) to give compounds **3a–c**. Next the solution was stirred at 180 °C and purified by column chromatography to obtaine the rearranged compounds **4a–c**. 

### 3.3. Preparation of 4-hydroxy-, and 7-hydroxycoumarin Derivatives via Ring Opening of Activated Aziridines

Synthesis of 4-hydroxy-, and 7-hydroxycoumarin derivatives with activated aziridines are shown in Scheme 2 and Scheme 3. Compound **5** was dissolved in dry acetone, CH_3_ONa and then added activated aziridine. The mixture was stirred at 130 °C, and dry mass was purified by column chromatography (CHCl3/EA = 9:1) for compounds **7** and **8**. Compound **9** was dissolved in dry acetone then added K2CO_3_ with same procedure for compounds **10** and **11**.

### 3.4. Preparation of Coumarin Amide Dimers

Synthesis of the desired amide dimer derivatives of coumarin acid with alkyldiamines is shown in scheme 4. The PyBOP coupling reagent was used to prepare a series of coumarin acid amide dimers under mild conditions. Compound **12** was dissolved in dry dimethylformamide (DMF) then added various alkyldiamines. When the mixtures were cooled PyBOP solution DMF and diisopropylethylamine (DIEA) was added. After the mixture was allowed to warm to room temperature and stirred for 18 h the residues were purified by column chromatography (CHCl_3_/acetone = 10:1) for compounds **14–21**.

*4-O-Acetyl-7-methoxycoumarin* (**3a**). M.p. 131~134 °C. ^1^H-NMR (CDCl_3_) δ: 2.40 (3H, s), 3.86 (3H, s), 6.82 (1H, d,*J* = 2.1 Hz), 6.84 (1H, dd,*J* = 7.6, 2.1 Hz), 7.47 (1H, d,*J* = 7.6 Hz), IR (KBr, cm^−1^) 1656 (C = O), 1721 (lactone).

*4-O-Acetyl-6-methylcoumarin* (**3b**). M.p. 128–130 °C. ^1^H-NMR (CDCl_3_) δ: 2.45 (3H, s), 2.47 (3H, s), 6.32 (1H, s), 7.26 (1H, d,*J* = 7.5 Hz), 7.46 (1H, dd,*J* = 7.5, 2.1 Hz), IR (KBr, cm^−1^) 1645, 1680 (C=O).

*4-O-Acetyl-6,7-dimethylcoumarin* (**3c**). M.p. 157–159 °C. ^1^H-NMR (CDCl_3_) δ: 2.35 (3H, s), 2.36 (3H, s), 2.42 (3H, s), 6.27 (1H, s), 7.13 (1H, s), 7.67 (1H, s). IR (KBr, cm^−1^) 1657, 1720 (C=O).

*4-O-Propionyl-7-methoxycoumarin* (**3a_1_**). M.p. 176–177 °C. ^1^H-NMR (CDCl_3_) δ: 1.53 (3H, t,*J* = 7.6 Hz), 2.45 (2H, q,*J* = 7.6 Hz), 3.86 (3H, s), 6.50 (1H, dd,*J* = 8.3, 2.4 Hz), 7.69 (1H,*J* = 8.3 Hz). IR (KBr) 1645, 1748 (C=O) cm^−1^.

*4-O-Propionyl-6-methylcoumarin* (**3b_1_**). M.p. 154–156 °C. ^1^H-NMR (CDCl_3_) δ: 1.22 (3H, t,*J* = 7.6 Hz), 2.43 (3H, s), 3.22 (2H, q,*J* = 7.6 Hz), 7.04 (1H, s), 7.20 (1H, d,*J* = 7.9 Hz), 7.47 (1H, dd,*J* = 7.9, 1.9 Hz), 7.84 (1H, s). IR (KBr, cm^−1^) 1634, 1701 (C=O).

*4-O-Propionyl-6,7-dimethylcoumarin* (**3c1**). M.p. 167–168 °C. ^1^H-NMR (CDCl_3_) δ: 1.32 (3H, t,*J* = 7.5 Hz), 2.31 (3H, s), 2.35 (3H, s), 2.72 (2H, q, *J* = 7.5 Hz), 6.41 (1H, s), 7.14 (1H, s), 7.30 (1H, s). IR (KBr, cm^−1^) 1646, 1711 (C=O). MS m/z (rel. int %): 246 (M)^+^ (14), 190 (51), 148 (78), 91 (20), 57 (100).

*4-O-Benzoyl-7-methoxycoumarin* (**3a_2_**). M.p. 147–148 °C. ^1^H-NMR (CDCl_3_) δ: 3.87 (3H, s), 6.42 (1H, s), 6.84 (1H, d, *J* = 2.2 Hz), 6.84 (1H, dd, *J* = 7.5, 2.2 Hz), 7.54 (1H, d, *J* = 7.5 Hz), 7.54 (2H, t, *J* = 7.5 Hz), 7.68 (1H, t, *J* = 7.5 Hz), 8.20 (2H, d, *J* = 7.5 Hz). IR (KBr, cm^−1^) 1640, 1710 (C=O). MS m/z (rel. int %): 296 (M)^+^ (2), 105 (1), 77 (40), 51 (20).

*4-O-Benzoyl-6-methylcoumarin* (**3b_2_**). M.p. 126–128 °C. ^1^H-NMR (CDCl_3_) δ: 2.41 (3H, s), 6.56 (1H, s), 7.29 (1H, d, *J* = 8.4 Hz), 7.39 (1H, dd, *J* = 8.4, 2.1 Hz), 7.44 (1H, d, *J* = 2.1 Hz), 7.58 (2H, t, *J* = 7.7 Hz), 7.72 (1H, t, *J* = 7.7 Hz), 8.24 (2H, d, *J* = 7.7 Hz). IR (KBr, cm^−1^) 1645, 1720 (C=O).

*4-O-Benzoyl-6,7-dimethylcoumarin* (**3c_2_**). M.p. 163–164 °C. ^1^H-NMR (CDCl_3_) δ: 2.26 (3H, d, *J* = 7.1 Hz), 2.33 (3H, d, *J* = 7.1 Hz), 6.46 (1H, s), 7.16 (1H, s), 7.35 (1H, s), 7.55 (2H, t, *J* = 7.9 Hz), 7.69 (1H, t, *J* = 7.9 Hz), 8.21 (2H, d, *J* = 7.9 Hz). IR (KBr, cm^−1^) 1665, 1728 (C=O).

*3-Acetyl-4-hydroxy-7-methoxycoumarin* (**4a**). M.p. 194–196 °C. ^1^H-NMR (CDCl_3_) δ: 2.73 (3H, s), 3.89 (3H, s), 6.72 (1H, d, *J* = 2.1 Hz), 6.85 (1H, dd, *J* = 8.9, 2.1 Hz), 7.92 (1H, d, *J* = 8.9 Hz). IR (KBr, cm^−1^) 1661, 1753 (C=O), 3442 (OH). LC/MS/MS (ESI) *m/z*, (rel. int. %): 234.9(M)^+^ (20), 217.3 (–OH, 100), 192.9 (–acetyl, 62), 151.1 (74).

*3-Acetyl-4-hydroxy-6-methylcoumarin* (**4b**). M.p. 156–159 °C. ^1^H-NMR (CDCl_3_) δ: 2.43 (3H, s), 2.78 (3H, s), 7.19 (1H, d, *J* = 8.4 Hz), 7.48 (1H, dd, *J* = 8.4, 1.5 Hz), 7.84 (1H, s). IR (KBr) 1661, 1753 (C=O), 3410 (OH). LC/MS/MS (ESI) *m/z*, (rel. int. %) 243.2 (M+Na) (80), 203.1 (M+Na−OH, 90), 135.1 (100).

*3-Acetyl-4-hydroxy-6, 7-dimethylcoumarin* (**4c**). M.p. 168–170 °C. ^1^H-NMR (CDCl_3_) δ: 2.36 (3H, s), 2.41 (3H, s), 2.79 (3H, s), 7.11 (1H, s), 7.50 (1H, s). IR (KBr, cm^−1^) 1631, 1723 (C=O), 3419 (OH). LC/MS/MS (ESI) *m/z*, (rel. int. %) 233.2 (M+H) (30), 215.1 (M+H−H_2_O), (100), 149.2 (60).

*3-Acetyl-4-hydroxy-7-methoxycoumarin* (**4a_1_**). M.p. 197–199 °C. ^1^H-NMR (CDCl_3_) δ: 1.32 (3H, t, *J* = 7.6 Hz), 2.72 (2H, q, *J* = 7.6 Hz), 3.86 (3H, s), 6.79 (1H, d, *J* = 2.2 Hz), 6.90 (1H, dd, *J* = 7.8, 2.2 Hz), 7.83 (1H, d, *J* = 7.8 Hz). IR (KBr, cm^−1^) 1653, 1763 (C=O), 3438 (OH). LC/MS/MS (ESI) *m/z*, (rel. int. %) 287.0 (M+K) (60), 272.1 (M+K−CH), (100), 257.3 (M+K−OCH_3_) (33), 244.1 (M+K−C_3_H_7_) (90).

*3-Propionyl-4-hydroxy-6-methylcoumarin* (**4b_1_**). M.p. 191–192 °C. ^1^H-NMR (CDCl_3_) δ: 1.37 (3H, t, *J* = 7.5 Hz), 2.47 (3H, s), 2.77 (2H, q, *J* = 7.1 Hz), 7.46 (1H, dd, *J* = 7.1, 2.1 Hz), 7.73 (1H, s), IR (KBr, cm^−1^) 1652, 1759 (C=O). LC/MS/MS (ESI) *m/z*, (rel. int. %) 271.2 (M+K)^+^ (40), 256.0 (M+K−CH_3_)^+^ (60), 203.2 (70), 135.0(100).

*3-Propionyl-4-hydroxy-6,7-dimethylcoumarin* (**4c_1_**). M.p. 201–202 °C. ^1^H-NMR (CDCl_3_) δ: 1.34 (3H, t, *J* = 7.6 Hz), 2.35 (3H, s), 2.37 (3H, s), 2.74 (2H, q, *J* = 7.6 Hz), 7.12 (1H, s), 7.65 (1H, s). IR (KBr, cm^−1^) 1652, 1730 (C=O), 3400 (OH). LC/MS/MS (ESI) *m/z*, (rel. int. %) 285.1 (M+K)^+^ (80), 270.3 (M+K−CH_3_)^+^, 255.1 (23), 227.0 (18), 217.2 (70), 149.1 (100), 105.0 (18).

*3-Benzoyl-4-hydroxy-7-methoxycoumarin* (**4a_2_**). M.p. 182–183 °C. ^1^H-NMR (CDCl_3_) δ: 3.89 (3H, s), 6.86 (1H, d, *J* = 2.3 Hz), 6.88 (1H, dd, *J* = 7.7, 2.3 Hz), 7.56 (1H, t, *J* = 7.7 Hz), 7.54 (2H, t, *J* = 7.8 Hz), 7.72 (1H, t, *J* = 7.8 Hz), 8.22 (2H, d, *J* = 7.8 Hz). IR (KBr, cm^−1^) 1650, 1714 (C=O), 3425 (OH).

*3-Benzoyl-4-hydroxy-6-methylcoumarin* (**4b_2_**). M.p. 176–178 °C. ^1^H-NMR (CDCl_3_) δ: 2.41 (3H, s), 7.29 (1H, d, *J* = 7.6 Hz), 7.40 (1H, dd, *J* = 7.6, 1.8 Hz), 7.46 (1H, s), 7.58 (2H, t, *J* = 7.7 Hz), 7.72 (1H, t, *J* = 7.7 Hz), 8.24 (2H, d, *J* = 7.7 Hz). IR (KBr, cm^−1^) 1655, 1724 (C=O), 3445 (OH).

*3-Benzoyl-4-hydroxy-6,7-dimethylcoumarin* (**4c_2_**). M.p. 197–198 °C. ^1^H-NMR (CDCl_3_) δ: 2.30 (3H, d, *J* = 7.1 Hz), 2.37 (3H, d, *J* = 7.1 Hz), 7.16 (1H, s), 7.38 (1H, s), 7.57 (2H, t, *J* = 7.7 Hz), 7.71 (1H, t, *J* = 7.7 Hz), 8.24 (2H, d, *J* = 7.7 Hz). IR (KBr, cm^−1^) 1665, 1728 (C=O), 3480 (OH).

*4-(N-Benzoylamino)ethoxycoumarin* (**7d**). M.p. 172–173 °C. ^1^H-NMR (CDCl_3_) δ: 4.01 (2H, t, *J* = 5.2 Hz), 4.32 (2H, t, *J* = 5.2 Hz), 5.71 (1H, s), 6.53 (1H, s), 7.28 (1H, d, *J* = 7.7 Hz), 7.32 (1H, t, *J* = 7.7 Hz). 7.43 (2H, t, *J* = 8.2 Hz), 7.44 (1H, t, *J* = 7.7 Hz), 7.52 (1H, t, *J* = 8.2 Hz), 7.78 (1H, d, *J* = 7.7 Hz), 7.83 (2H, d, *J* = 8.2 Hz). IR (KBr, cm^−1^) 1653, 1730 (C=O), 3223 (NH). MS *m/z* (rel. int. %) 309 (2), 148 (91), 105 (100), 77 (95). HR-FAB-MS (EI, 80 eV) *m/z*, calculated for C_18_H_15_NO_4_ 309.1001, found 309.1000.

*4-[(N-Benzoylamino)-2-propoxyl]coumarin* (**7e**). M.p. 193–194 °C. ^1^H-NMR (CDCl_3_) δ: 1.41 (3H, d, *J* = 6.1 Hz), 3.79 (1H, dd, *J* = 6.4, 10.2 Hz), 3.94 (1H, dd, *J* = 3.8, 6.3, 10.2 Hz), 4.88 (1H, dd, *J* = 6.4, 10.2 Hz), 5.78 (1H, s), 7.25 (1H, d, *J* = 7.3 Hz), 7.27 (1H, t, *J* = 7.3 Hz), 7.39 (1H, t, *J* = 7.3 Hz), 7.42 (2H, t, *J* = 7.8 Hz), 7.78 (2H, d, *J* = 7.8 Hz). IR (KBr, cm^−1^) 1635, 1735 (C=O), 3214 (NH). MS *m/z*, (rel. int. %) 323 (1), 174 (25), 148 (48), 59 (100).

*4-[(N-Benzoylamino)-2-methyl-2-propoxy]coumarin* (**7f**). M.p. 198–200 °C. ^1^H-NMR (CDCl_3_) δ: 1.38 (3H, s), 3.88 (2H, d, *J* = 6.3 Hz), 5.87 (1H, s), 6.52 (1H, t-like), 7.27 (1H, *J* = 7.6 Hz), 7.29 (1H, t, *J* = 7.6 Hz), 7.41 (1H, t, *J* = 7.6 Hz), 7.44 (2H, t, *J* = 7.9 Hz), 7.75 (1H, d, *J* = 7.6 Hz), 7.78 (1H, t, *J* = 7.9 Hz), 7.79 2H, d, *J* = 7.9 Hz). IR (KBr, cm^−1^) 1648, 1742 (C=O), 3300 (NH). MS *m/z*, (rel. int. %) 337 (M)^+^ (1), 212 (21), 141 (25), 77 (100), 70 (90). HR-FAB-MS (EI, 80 eV) *m/z*, calculated for C_2_0H_19_NO_4_ 337.1314, found 337.1322.

*4-[(N-Ethylcarbamyl)ethyl]coumarin* (**7g**). M.p. 123–124 °C. ^1^H-NMR (CDCl_3_) δ: 1.24 (3H, t, *J* = 6.5 Hz), 3.71 (2H, d, *J* = 5.1 Hz), 4.14 (2H, t, *J* = 5.1 Hz), 4.17 (2H, q, *J* = 6.5 Hz), 5.03 (1H, s), 5.68 (1H, s), 7.28 (1H, d, *J* =7.9 Hz), 7.31 (1H, t, *J* = 7.9 Hz), 7.54 (1H, t, *J* = 7.9 Hz), 7.80 (1H, d, *J* = 7.9 Hz). IR (KBr, cm^−1^) MS *m/z*, (rel. int. %) 277 (10), 116 (80), 88 (100).

*4-[(N-Benzosulfonyl)-1,1-dimethylethyl]coumarin* (**7h**). M.p. 169–171 °C. ^1^H-NMR (CDCl_3_) δ: 1.61 (6H, s), 3.31 (2H, d, *J* = 6.8 Hz), 5.72 (1H, s), 7.23–7.29 (2H, dd, *J* = 8.1, 8.2 Hz), 7.54 (3H, dd, *J* = 1.3, 7.4 Hz), 7.62 (2H, ddd, *J* = 1.2, 7.4, 7.8 Hz), 7.86 (2H, dd-like, *J* = 1.1, 7.2 Hz). IR (KBr, cm^−1^) 1638, 1692 (C=O), 3200 (NH). HR-FAB-MS (EI, 80 eV) *m/z*, calculated for C_19_H_19_NO_5_S 373.0984, found 373.0986.

*4-[(N-p-Tolylsulfonyl)-2-methylethyl]coumarin* (**8i**). M.p. 154–156 °C. ^1^H-NMR (CDCl_3_) δ: 1.28 (3H, d, *J* = 6.8 Hz), 2.39 (3H, s), 3.83 (1H, m), 3.96 (2H, d, *J* = 3.8 Hz), 4.93 (1H, d, *J* = 7.7 Hz), 5.48 (1H, s), 7.21 (1H, d, *J* = 7.6 Hz), 7.23 (1H, t, *J* = 7.6 Hz), 7.29 (2H, d, *J* = 8.2 Hz), 7.53 (1H, t, *J* = 7.6 Hz), 7.65 (1H, d, *J* = 7.6 Hz), 7.72 (2H, d, *J* = 8.2 Hz). IR (KBr, cm^−1^) 1620, 1693 (C=O), 3172 (NH). MS *m/z*, (rel. int. %) 373 (10), 218 (25), 155 (46), 91 (100). HR-FAB-MS (EI, 80 eV) *m/z*, calculated for C_19_H_19_NO_5_S 373.0984, found 373.0986.

*4-[(N-p-Tolylsulfonyl)-1,1-dimetylethyl]coumarin* (**8j**). M.p. 187–190 °C. ^1^H-NMR (CDCl_3_) δ: 1.43 (3H, s), 2.23 (3H, s), 3.89 (2H, s), 4.97 (1H, s), 5.85 (1H, s), 7.07 (1H, d, *J* = 7.3 Hz), 7.28 (1H, t, *J* = 7.3 Hz), 7.53 (1H, t, *J* = 7.3 Hz), 7.65 (2H, d, *J* = 7.5 Hz), 7.68 (1H, d, *J* = 7.3 Hz), 7.73 (2H, d, *J* = 7.5 Hz). IR (KBr, cm^−1^) 1620, 1683 (C=O), 3425 (NH). MS *m/z*, (rel. int. %) 387 (M)^+^ (2), 212 (25), 155 (30), 91 (100), 70 (70).

*7-[(N-p-Tosyl)-2,2-dimethylethyloxy]coumarin* (**10k**). M.p. 220–221 °C. ^1^H-NMR (CDCl_3_) δ: 1.36 (6H, s), 2.45 (3H, s), 3.76 (2H, s), 4.91 (1H, s), 6.72 (1H, d, *J* = 9.5 Hz), 6.62 (1H, bs), 6.76 (1H, dd, *J* = 8.5, 1.8 Hz), 7.18 (2H, d, *J* = 7.9 Hz), 7.36 (1H, d, *J* = 7.9 Hz), 7.63 (1H, d, *J* = 9.5 Hz), 7.74 (2H, d, *J* = 8.0 Hz). IR (KBr, cm^−1^) 1692 (C=O), 3406 (NH).

*7-[(N-p-Tosyl)-2-methylethyloxy]coumarin* (**10l**).M.p. 110–111 °C. ^1^H-NMR (CDCl_3_) δ: 1.15 (3H, s), 2.26 (3H, s), 3.62 (1H, t-like, *J* = 5.5 Hz), 3.73 (1H, dd, *J* = 5.1, 9.3 Hz), 3.81 (1H, dd, *J* = 4.9, 9.2 Hz), 5.87 (1H, d, *J* = 7.6 Hz), 6.12 (1H, d, *J* = 9.2 Hz), 6.50 (1H, bs), 6.58 (1H, d, *J* = 8.3 Hz), 7.13 (2H, d, *J* = 7.7 Hz), 7.21 (1H, d, *J* = 8.5 Hz), 7.56 (1H, d, *J* = 9.5 Hz), 7.67 (2H, d, *J* = 8.0 Hz). IR (KBr, cm^−1^) 1718 (C=O), 3421 (NH). 

*7-[(N-Phenylaminylcarboxamido)ethyloxy]coumarin* (**10m**). M.p. 164–165 °C. ^1^H-NMR (CDCl_3_) δ: 3.53 (2H, t, *J* = 5.5 Hz), 4.07 (2H, t, *J* = 5.3 Hz), 6.14 (1H, d, *J* = 9.5 Hz), 6.24 (1H, t, *J* = 5.6 Hz), 6.78 (1H, d, *J* = 2.3 Hz), 6.82 (1H, dd, *J* = 2.8, 8.6 Hz), 6.83 (1H, ddd, *J* = 1.7, 2.7, 6.9 Hz), 7.13 (2H, dd, *J* = 2.3, 8.0 Hz), 7.30 (2H, d, *J* = 8.0 Hz), 7.39 (1H, d, *J* = 8.6 Hz), 7.68 (1H, d, *J* = 8.1 Hz), 8.23 (1H, s). IR (KBr, cm^−1^) 1660, 1701 (C=O), 3312 (NH).

*7-[(N-Benzoyl)-1-methylethyloxy]coumarin* (**10n**). M.p. 135–136 °C. ^1^H-NMR (CDCl_3_) δ: 1.45 (3H, d, *J* = 6.8 Hz), 4.15 (2H, ddd, *J* = 4.5, 9.3, 12.4 Hz), 4.65 (1H, dd, *J* = 3.5, 7.1 Hz), 6.25 (1H, d, *J* = 9.5 Hz), 6.85 (1H, d, *J* = 2.2 Hz), 6.89 (1H, dd, *J* = 7.8, 2.3 Hz), 7.38 (1H, d, *J* = 8.6 Hz), 7.44 (2H, dd, *J* = 7.8, 2.1 Hz), 7.50 (1H, d, *J* = 7.3 Hz), 7.62 (1H, d, *J* = 9.5 Hz), 7.77 (2H, d, *J* = 7.5 Hz). IR (KBr, cm^−1^) 1662, 1708 (C=O), 3330 (NH).

*7-[(2-Adamantanecarboxamido)ethyloxy]coumarin* (**10o**). Oil. ^1^H-NMR (CDCl_3_) δ: 1.11 (3H, s), 1.72 (6H, dd, *J* = 12.3, 26.9 Hz), 1.86 (6H, d, *J* = 2.4 Hz), 2.05 (3H, s), 3.69 (2H, dd, *J* = 5.5, 10.8 Hz), 4.10 (2H, t, *J* = 5.1 Hz), 6.03 (1H, s), 6.26 (1H, d, *J* = 9.5 Hz). 6.82 (1H, d, *J* = 2.2 Hz), 6.85 (1H, dd, *J* = 8.6, 2.4 Hz), 7.38 (1H, d, *J* = 8.5 Hz), 7.63 (1H, d, *J* = 9.5 Hz). IR (KBr, cm^−1^) 1663, 1705 (C=O), 3282 (NH).

*7-[(N-p-Phenylbenzoyl)-ethyloxy]coumarin* (**10p**). M.p. 216–217 °C. ^1^H-NMR (CDCl_3_) δ: 3.95 (2H, dd, *J* = 5.5, 10.8 Hz), 4.24 (2H, t, *J* = 5.1 Hz), 6.86 (1H, d, *J* = 2.3 Hz), 6.88 (1H, dd, *J* = 8.4, 2.3 Hz), 7.39 (2H, d, *J* = 8.4 Hz), 7.46 (2H, t, *J* = 7.6 Hz), 7.61 (2H, t, *J* = 4.5 Hz), 7.63 (1H, d, *J* = 9.5 Hz), 7.67 (2H, d, *J* = 8.3 Hz), 7.87 (2H, d, 8.3 Hz). IR (KBr, cm^−1^) 1656, 1697 (C=O), 3356 (NH).

*7-[(N-Benzoyl)-1,1-dimethylethyloxy]coumarin* (**10q**). M.p. 104–105 °C. ^1^H-NMR (CDCl_3_) δ: 1.40 (6H, s), 3.71 (2H, d, *J* = 6.0 Hz), 6.27 (1H, d, *J* = 9.5 Hz), 6.76 (1H, d, *J* = 4.2 Hz), 6.88 (1H, d, *J* = 2.2 Hz), 6.89 (1H, dd, *J* = 8.3, 2.2 Hz), 7.36 (1H, d, *J* = 8.4 Hz), 7.43 ( 2H, t, *J* = 7.5 Hz), 7.49 (1H, t, *J* = 7.3 Hz), 7.63 (1H, d, *J* = 9.5 Hz), 7.81 (2H, d, *J* = 7.4 Hz). IR (KBr, cm^−1^) 1651, 1692 (C=O), 3366 (NH).

*7-[(N-Ethylcarbamyl)ethyloxy]coumarin* (**10r**). M.p. 115–116 °C. ^1^H-NMR (CDCl_3_) δ: 1.25 (3H, t, *J* = 6.8 Hz), 3.62 (2H, d, *J* = 5.1 Hz), 4.09 (1H, t, *J* = 5.0 Hz), 4.14 (1H, t, *J* = 6.8 Hz), 5.10 (1H, bs), 6.26 (1H, d, *J* = 9.6 Hz), 6.80 (1H, d, *J* = 1.8 Hz), 6.84 (1H, dd, *J* = 8.6, 2.2 Hz), 7.38 (1H, d, *J* = 8.6 Hz), 7.63 (1H, d, *J* = 9.5 Hz). IR (KBr, cm^−1^) 1677, 1733 (C=O), 3292 (NH).

*7-[(N-p-Tosyl)-1,1-dimethylethyloxy]coumarin* (**11k**). M.p. 177–178 °C. ^1^H-NMR (CDCl_3_) δ: 1.36 (6H, s), 2.45 (3H, s), 3.12 (2H, d, *J* = 6.4 Hz), 4.88 (1H, t, *J* = 5.8 Hz), 6.32 (1H, d, *J* = 9.5 Hz), 6.80 (2H, d, *J* = 7.3 Hz), 7.35 (3H, dd, *J* = 7.4, 5.5 Hz), 7.64 (1H, d, *J* = 9.5 Hz), 7.76 (2H, d, *J* = 8.1 Hz). IR (KBr, cm^−1^) 1721 (C=O), 3416 (NH)

*Bis(3-coumarin) propylenediamide* (**14**). M.p. 264–265 °C. ^1^H-NMR (CDCl_3_) δ: 1.98 (2H, dd, *J* = 6.5, 13.3 Hz), 3.58 (2H, dd, *J* = 6.4, 12.8 Hz), 7.38 (2H, dd, *J* = 7.9, 7.6 Hz), 7.67 (2H, ddd, *J* = 1.1, 7.4, 8.4 Hz), 8.92 (1H, bs), 9.01 (1H, s). ^13^C-NMR δ 29.61 (CH_2_CN), 37.40 (2×CCH_2_N), 116.62 (C-4,4'), 118.55 (C-8,8'), 118.68 (C-5,5'), 125.22 (C-6,6'), 129.77 (C-7,7'), 133.94 (C-9,9'), 148.31 (C-3,3'), 154.46 (C-10,10'), 161.38 (OCN), 161.82 (O=CO). IR (KBr, cm^−1^) 1658, 1705 (C=O), 3341 (NH). HR-FAB-MS (EI, 80 eV) *m/z*, calculated for C_23_H_18_N_2_O_6_ 418.1165, found 418.1163.

*Bis(3-coumarin)butamethylenediamide* (**15**). M.p. 250–251 °C. ^1^H-NMR (CDCl_3_) δ: 1.75 (2H, t, *J* = 2.8 Hz), 3.52 (2H, d, *J* = 5.8 Hz), 7.39 (2H, dd, *J* = 8.8, 7.5 Hz), 7.68 (2H, ddd, *J* = 8.8, 7.7, 1.4 Hz), 8.86 (1H, bs, NH), 8.91 (1H, s, H-4).^13^C-NMR δ: 27.02 (2×NCCH_2_), 39.47 (2×NCH_2_C), 116.61 (C-4,4'), 118.51 (C-8,8'), 118.67 (C-5,5'), 125.25 (C-6,6'), 129.79 (C-7,7'). 133.96 (C-9,9'), 148.31(C-3,3'), 154.41 (C-10,10'), 161.41 (OCN), 161.48 (O=CO). IR (KBr, cm^−1^) 1658, 1710 (C=O), 3337 (NH). HR-FAB-MS (EI, 80 eV) *m/z*, calculated for C_24_H_20_N_2_O_6_ 432.1321, found 432.1471.

*Bis(3-coumarin)pentamethylenediamide* (**16**). M.p. 221–222 °C. ^1^H-NMR (CDCl_3_) δ: 1.52 (2H, ddd, *J* = 6.9, 8.4, 12.1 Hz), 1.70 (4H, dd, *J* = 7.3, 14.7 Hz), 3.49 (4H, dd, *J* = 13.1, 6.9 Hz, 2N-CH_2_), 7.38 (4H, dd, *J* = 7.7, 8.8 Hz), 7.67 (4H, dd, *J* = 7.7, 8.4 Hz), 8.82 (2H, bs, NH), 8.90 (2H, s, H-4,4'). ^13^C-NMR δ: 24.38 (NCCCH_2_), 29.08 (2×NCCH_2_), 39.67 (2×CH_2_), 116.61 (C-4,4'), 118.63 (C-8,8'), 118.72 (C-5,5'), 125.22 (C-6,6'), 129.77 (C-7,7'), 133.88 (C-9,9'), 148.19 (C-3,3'), 154.43 (C-10,10'), 161.45 (OCN), 161.47 (OCO). IR (KBr, cm^−1^) 1654, 1702 (C=O). HR-FAB-MS (EI, 80 eV) *m/z*, calculated for C_25_H_22_N_2_O_6_ 446.1478, found 446.1477.

*Bis(3-coumarin)hexamethylenediamide* (**17**). M.p. 212–213 °C. ^1^H-NMR (CDCl_3_) δ: 1.46 (4H, t-like, *J* = 7.1, 12.2 Hz), 1.66 (4H, t-like *J* = 6.7, 13.5 Hz), 3.47 (4H, dd, *J* = 6.8, 13.1 Hz), 7.38 (4H, dd, *J* = 7.6, 8.5 Hz), 7.68 (4H, dd, *J* = 7.2, 8.5 Hz), 8.81 (2H, bs, NH), 8.91 (2H, s, H-4,4'). ^13^C-NMR δ: 26.71 (2×NCCCH_2_), 29.31 (2×NCCH_2_), 39.80 (2×NCH_2_), 116.61 (C-4,4'), 118.65 (C-8,8'), 118.72 (C-5,5'), 125.22 (C-6,6'), 129.76 (C-7,7'), 133.88 (C-9,9'), 148.17 (C-3,3'), 154.42 (C-10,10'), 161.41 (OCN),161.48 (OCO). IR (KBr, cm^−1^) 1668, 1712 (C=O), 3341 (NH). HR-FAB-MS (EI, 80 eV) *m/z*, calculated for C_26_H_24_N_2_O_6_ 460.1634, found 460.1640.

*Bis(3-coumarin)heptamethylenediamide* (**18**). M.p. 198–199 °C. ^1^H-NMR (CDCl_3_) δ: 1.42 (6H, s, NCC(CH_2_)_3_), 1.64 (4H, t-like, *J* = 6.2, 12.9 Hz, NC(CH_2_)_2_), 3.47 (4H, dd, *J* = 7.0, 13.1 Hz, N(CH_2_)_2_), 7.38 (4H, dd, *J* = 7.5, 9.2 Hz, H-6,6',8,8'), 7.66 (4H, dd, *J* = 1.1, 7.7, 9.0 Hz, H-5,5',7,7'), 8.80 (2H, bs, 2×NH), 8.91 (2H, s, H-4,4'). ^13^C-NMR δ: 26.90 (NCCCCH_2_), 28.94 (NCCCH_2_), 29.32 (NCCH_2_), 39.85 (NCH_2_), 116.0 (C-4,4'), 118.65 (C-8,8'), 118.72 (C-5,5'), 125.21 (C-6,6'), 129.75 (C-7,7'), 133.87 (C-9,9'), 148.16 (C-3,3'), 154.41 (C-10,10'), 161.37 (CON), 161.47 (COO). IR (KBr, cm^−1^) 1662, 1704 (C=O), 3368 (NH). MS (rel.int. %) 475.384 (M^+^+H) (40), 191.041 (100), 173.029 (80). HR-FAB-MS (Ei, 80 eV) *m/z*, calculated for C_27_H_26_N_2_O_6_ 474.1791, found 474.2761.

*Bis(3-coumarin)octamethylenediamide* (**19**). M.p. 199–200 °C. ^1^H-NMR (CDCl_3_) δ: 1.31 (8H, t-like, *J* = 3.8, 7.1 Hz), 1.63 (4H, dd, *J* = 7.1, 14.0 Hz), 3.46 (4H, dd, *J* = 7.0, 13.1 Hz, 2×NCH_2_), 7.38 (4H, dt, *J* = 1.4, 7.5, 8.4 Hz, H-6,6',8,8'), 7.67 (4H, dddd, *J* = 1.0, 1.2, 8.6,8.9 Hz, H-5,5',7,7'), 8.80 (2H, bs, 2×NH), 8.91 (2H, s, H-4,4'). ^13^C-NMR δ: 26.94 (NCCCCH_2_), 29.16 (NCCCH_2_), 29.36 (NCCH2), 39.90 (NCH_2_), 116.61 (C-4,4'), 118.67 (C-8,8'), 118.72 (C-5,5'), 125 22 (C-6,6'), 129.76 (C-7,7'), 133.88 (C-9,9'), 148.14 (C-3,3'), 154.42 (C-10,10'), 161.37 (OCN), 161.49 (OCO). IR (KBr, cm^−1^) 1665, 1710 (C=O), 3342 (NH). HR-FAB-MS (EI, 80 eV) *m/z*, calculated for C_28_H_28_N_2_O_6_ 488.1947, found 488.1951.

*Bis(3-coumarin)decamethylenediamide* (**20**). M.p. 203–204 °C. ^1^H-NMR (CDCl_3_) δ: 1.37 (12H, t-like, *J* = 5.9, 8.9 Hz), 1.63 (4H, dt, *J* = 7.4, 14.3 Hz), 3.46 (4H, dd, *J* = 6.9, 13.2 Hz, NCH_2_), 7.38 (4H, *J* = 7.5, 8.1 Hz, H-6,6',8,8'), 7.67 (4H, dd, *J* = 7.7, 8.6 Hz, H-5,5'7,7'), 8.80 (2H, bs, NH), 8.91 (2H, s, H-4,4'). ^13^C-NMR δ: 27.01, 29.26, 29.38, 29.43, 39.96 (NCH_2_), 116.61 (C-4,4'), 118.68 (C-8,8'), 118.73 (C-5,5'), 125.22 (C-6,6'), 129.76 (C-7,7'), 133.88 (C-9,9'), 148.14 (C-3,3'), 154.42 (C-10,10'), 161.39 (OCN), 161.49 (OCO). IR (KBr, cm^−1^) 1647, 1688 (C=O), 3310 (NH). LCMS 517.305 (M^+^+H), HR-FAB-MS (EI, 80eV) *m/z*, calculated for C_30_H_32_N2O_6_ 512.2260, found 516.2051.

*Bis(3-coumarin)dodecamethylenediamide* (**21**). M.p. 172–173 °C. ^1^H-NMR (CDCl_3_) δ: 1.36 (16H, ddd, *J* = 6.6,7.5,13.5 Hz), 1.63 (4H, dt, *J* = 7.5, 14.4 Hz), 3.45 (4H, dd, *J* = 7.0, 13.1 Hz, 2×NCH_2_), 7.38 (4H, dt, *J* = 7.5, 8.4 Hz, H-6,6',8,8'), 7.68 (4H, dddd, *J* = 1.3, 5.7, 7.6, 8.7 Hz, H-5,5',7,7'), 8.79 (2H, bs, NH), 8.91 (2H, s, H-4,4'). ^13^C-NMR δ: 27.03, 29.30, 29.38, 29.51, 29.54, 39.96 (NCH_2_), 116.61 (C-4,4'), 118.68 (C-8,8'), 118.73 (C-5,5'), 125.22 (C-6,6'), 129.76 (C-7,7'), 133.88 (C-9,9'), 148.14 (C-3,3'), 154.42 (C-10,10'), 161.39 (OCN), 161.49 (OCO). IR (KBr, cm^−1^) 1664, 1707 (C=O), 3362 (NH). HR-FAB-MS (EI, 80eV) *m/z*, calculated for C_32_H_36_N_2_O_6_ 544.2573, found 544.2572. 

## 4. Conclusions

Synthesis of coumarin derivatives via the ring-opening reaction with activated aziridines has not been reported previously. The reaction of the coumarin anion with *N*-benzoyl- or *N*-sulfonylaziridines **6** yielded amidoethylated 7- or 4-hydroxycoumarins **7**, **8**, **9** and **10**. The final results depend on the substitution of the *N*-acyl or *N*-sulfonyl groups on the acyl group, so the benzoyl and the coumarin anions will attack from an abnormal position to provide the major products **7d–f**, **10n**, and **10q**. The main products resulted from normal ring-opening of the activated aziridines **7h**, **8i**, **8j**, **10k–l**, and **11k**. It was assumed that compounds **7**, **8**, **10**, **11**, and their analogues were formed by the nucleophilic attack of acylaziridines via an S_N_2-like ring opening mechanism. In our previous work, when PyBOP (**13**) used as a coupling reagent to synthesize natural amide dimers, relatively high yields were obtained. In general, for the formation of amide or dimer chains, the reaction temperature had a great influence on the structural conformation. From NMR spectroscopic analysis, the intermediate, coumarin-triazobenzene, displayed rearrangement characteristics. The antimicrobial tests performed on coumarin derivatives **3c**, **3c_1_**, **4a_2_**, **4b**, **4c**, and **7f** confirmed the better potential activities of these compounds against Gram-positive rather than Gram-negative bacteria. Compound **7f** was the most potent of these tested compounds against *B. subtilis*, with a MIC value of 8 μg/mL, while the coumarin amide dimers **14–21** displayed lower antibacterial activities (the MICs are >128 to 256).
